# Implementation of the Ebola Virus Persistence in Ocular Tissues and Fluids (EVICT) study: Lessons learned for vision health systems strengthening in Sierra Leone

**DOI:** 10.1371/journal.pone.0252905

**Published:** 2021-07-09

**Authors:** Jessica G. Shantha, Ian Crozier, Colleen S. Kraft, Donald G. Grant, Augustine Goba, Brent R. Hayek, Caleb Hartley, Kayla G. Barnes, Timothy M. Uyeki, John Schieffelin, Robert F. Garry, Daniel G. Bausch, Paul E. Farmer, John G. Mattia, Matthew J. Vandy, Steven Yeh, EVICT Study Investigators

**Affiliations:** 1 Emory Eye Center, Emory University School of Medicine, Atlanta, Georgia, United States of America; 2 National Institute for Allergy and Infectious Disease, Bethesda, Maryland, United States of America; 3 Department of Pathology and Laboratory Medicine, Emory University Serious Communicable Disease Unit, Atlanta, Georgia, United States of America; 4 Kenema Government Hospital Lassa Hemorrhagic Fever Laboratory, Kenema, Sierra Leone; 5 Department of Organismic and Evolutionary Biology, Harvard University, Broad Institute of MIT and Harvard, Cambridge, Massachusetts, United States of America; 6 Centers for Disease Control and Prevention, Atlanta, Georgia, United States of America; 7 Tulane University School of Medicine, New Orleans, Louisiana, United States of America; 8 United Kingdom Public Health Rapid Support Team (UK-PHRST), London School of Tropical Medicine and Hygiene, Public Health England, London, United Kingdom; 9 Partners in Health, Boston, Massachusetts, United States of America; 10 Lowell and Ruth Gess Eye Hospital, Freetown, Sierra Leone; 11 Ministry of Health and Sanitation, National Eye Programme, Freetown, Sierra Leone; 12 Emory Global Health Institute, Emory University, Atlanta, Georgia, United States of America; 13 Truhlsen Eye Institute, University of Nebraska Medical Center, Omaha, Nebraska, United States of America; University Hospitals Cleveland, UNITED STATES

## Abstract

**Background:**

Following the West African Ebola virus disease (EVD) outbreak of 2013–2016 and more recent EVD outbreaks in the Democratic Republic of Congo, thousands of EVD survivors are at-risk for sequelae including uveitis, which can lead to unremitting inflammation and vision loss from cataract. Because of the known risk of Ebola virus persistence in ocular fluid and the need to provide vision-restorative, safe cataract surgery, the Ebola Virus Persistence in Ocular Tissues and Fluids (EVICT) Study was implemented in Sierra Leone. During implementation of this multi-national study, challenges included regulatory approvals, mobilization, community engagement, infection prevention and control, and collaboration between multiple disciplines. In this report, we address the multifacted approach to address these challenges and the impact of implementation science research to address an urgent clinical subspecialty need in an outbreak setting.

**Methodology/Principal findings:**

Given the patient care need to develop a protocol to evaluate ocular fluid for Ebola virus RNA persistence prior to cataract surgery, as well as protocols to provide reassurance to ophthalmologists caring for EVD survivors with cataracts, the EVICT study was designed and implemented through the work of the Ministry of Health, Sierra Leone National Eye Programme, and international partnerships. The EVICT study showed that all 50 patients who underwent ocular fluid sampling at 19 and 34 months, respectively, tested negative for Ebola virus RNA. Thirty-four patients underwent successful cataract surgery with visual acuity improvement. Here we describe the methodology for study implementation, challenges encountered, and key issues that impacted EVD vision care in the immediate aftermath of the EVD outbreak. Key aspects of the EVICT study included defining the pertinent questions and clinical need, partnership alignment with key stakeholders, community engagement with EVD survivor associations, in-country and international regulatory approvals, study site design for infection prevention and control, and thorough plans for EVD survivor follow-up care and monitoring. Challenges encountered included patient mobilization owing to transportation routes and distance of patients in rural districts. Strong in-country partnerships and multiple international organizations overcame these challenges so that lessons learned could be applied for future EVD outbreaks in West and Central Africa including EVD outbreaks that are ongoing in Guinea and Democratic Republic of Congo.

**Conclusions/Significance:**

The EVICT Study showed that cataract surgery with a protocol-driven approach was safe and vision-restorative for EVD survivors, which provided guidance for EVD ophthalmic surgical care. Ophthalmologic care remains a key aspect of the public health response for EVD outbreaks but requires a meticulous, yet partnered approach with international and local in-country partners. Future efforts may build on this framework for clinical care and to improve our understanding of ophthalmic sequelae, develop treatment paradigms for EVD survivors, and strengthen vision health systems in resource-limited settings.

## Introduction

The West African Ebola virus disease (EVD) outbreak from 2013–2016 led to the largest EVD survivor cohort, predominantly within the most affected countries of Sierra Leone, Liberia, and Guinea [1,2]. In the aftermath of the West African outbreak, EVD survivor sequelae were increasingly recognized with findings that included uveitis, arthritis, mental health disorders, abdominal pain, and the risk of Ebola virus (EBOV) persistence in immune privileged organs [3–7]. Three additional EVD outbreaks have occurred in the Democratic Republic of Congo (DRC) during 2017–2020, including an outbreak in eastern DRC that resulted in 3,200 cases and over 2,000 deaths [8]. During February 2021, EVD outbreaks once again emerged in Guinea and in Democratic Republic of Congo, emphasizing the mandate to continue to understand the clinical implications of EVD, including EVD survivor sequelae and clinical research methodology.The thousands of EVD survivors within West Africa and the DRC emphasize the importance to broaden our understanding of the spectrum of clinical findings, mechanism of disease, and management algorithms for treating EVD sequelae. While advances have been made since the West African EVD outbreak with the availability of a licensed Ebola vaccine and proven therapies for EVD [9–11], further development of care protocols and consensus for EVD survivor health care remains a key issue.

Uveitis, an ocular inflammatory disease with the potential for vision compromise and blindness if untreated, has been identified with a particularly high prevalence in EVD survivors, ranging between 13–34% in prior retrospective series [4,12–14]. Recently, the National Institutes of Health-funded PREVAIL Study, a prospective longitudinal natural cohort study of EVD survivors in Liberia, identified a 26% prevalence of uveitis, that increased to 33% at one-year follow-up. The prevalence of uveitis in EVD survivors was higher than in controls who were close-contacts of EVD cases, but notably, the control group had a higher prevalence of uveitis at baseline than reported in other epidemiologic studies (i.e. 12% prevalence of uveitis in controls at baseline and 15% at 1-year) [3]. Among EVD survivors who developed uveitis in Liberia, we observed that nearly 40% of patients with uveitis were blind due to a variety of complications including cataract, retinal scarring involving the macula, and optic nerve disease [4].

Cataract has been observed in 5–10% of EVD survivors [3,4]. However, vision loss due to cataract is reversible and presents an opportunity for vision restoration to improve work productivity, enhance an individual’s activities of daily living, and promote socialization. The potential reduction of stigmata in EVD survivors where dense white cataract may be visible is a strong consideration for the well-being of the individual. Given that Ebola virus (EBOV) persistence had been identified in ocular fluid in association with sight-threatening panuveitis [6], there has been uncertainty regarding the risk of EBOV persistence in eye fluid and the best approach for invasive eye surgery. These considerations were particularly important for ophthalmologists, eye care nurses and providers to safely restore vision for EVD survivors through eye surgery with a measured approach.

To address these questions, we designed the **E**bola **Vi**rus Persistence in O**c**ular **T**issues and Fluids (EVICT) Study [15]. The purpose of the EVICT Study was to develop a protocol-driven approach to cataract surgery, staged to first assess ocular fluid for evidence of EBOV RNA persistence by RT-PCR, followed by cataract surgery for survivors whose ocular fluid tested negative for EBOV RT-PCR. During the EVICT study, fifty patients were enrolled and underwent ocular fluid sampling to evaluate EBOV RNA persistence in ocular fluid. All patients tested negative for EBOV RNA by RT-PCR in the aqueous humor (n = 49) or vitreous humor (n = 1) at a median of 19 months in Phase 1 of ocular fluid sampling and 34 months after EVD diagnosis in Phase 2 of ocular fluid sampling. Thirty-four patients eventually underwent manual small incision cataract surgery (MSICS) with improvement in median preoperative visual acuity from only hand motions to 20/30 at three-month postoperative follow-up (p<0.001) [15]. The clinical details are described in prior work.

Herein, we describe the implementation of this study including the formal research design, partnerships, implementation and lessons learned through vision research in a resource-limited setting. During the development and implementation of the EVICT study, the imperative to restore vision safely was aligned with research questions including clinical and postoperative evaluations and coordinated with advanced laboratory testing with Sierra Leone and international partners. Prior reports have documented some of the challenges of performing research in outbreak settings for reasons including supply shortages, insufficient health personnel, potential for EBOV infections in health care personnel and a depleted workforce, as well as systems of care conducive to both clinical care and research [16–18]. As research questions arise in emergent outbreak settings, a thoughtful approach to partnerships with local health care providers, identification of key clinical research questions, community engagement, rigorous study design, and implementation in resource-limited settings is needed.

Our description of the detailed implementation of the EVICT Study aims to inform ophthalmologists, infectious disease specialists and public health specialists regarding the unique challenges, lessons learned, and preparedness measures for EVD outbreaks and emerging infectious disease outbreaks with ophthalmic sequelae. Beyond the clinical care needs for EVD survivors, the implementation of the EVICT Study also provides key learnings for preparedness measures for clinical research and the provision of subspecialty care, invasive procedures and considerations for assessing immune privileged sites (e.g. eye, central nervous system, reproductive organs) that may harbor infectious pathogens.

## Methods

### Partnership alignment: A reflection of momentum from the acute EVD outbreak

Prior to initiation of the EVICT Study, the Ministry of Health and Sanitation (MOHS), Sierra Leone National Eye Programme, in partnership with the World Health Organization, Partners in Health, Emory University, GOAL, Save the Children and Medecins sans Frontieres organizations (Belgium, France, Switzerland) organized and implemented the National Eye Program screening program to perform ophthalmic examinations on a countrywide basis [19,20]. This was coordinated with the Comprehensive Programme of EVD Survivors (CPES) through the Program Implementation Unit of MOHS. The Sierra Leone Association of Ebola Survivors (SLAES) was organized during the acute EVD outbreak to advocate for their membership for systemic and ophthalmic health services, and SLAES played a key leadership and coordination role throughout the National Eye Program and the EVICT Study. Through the National Eye Screening program, which evaluated >3500 EVD survivors throughout Sierra Leone, individuals with uveitis were diagnosed and treatment was initiated. In addition, EVD survivors with cataract were identified for later screening in the EVICT Study. Moreover, EVD survivors who needed specialty care (e.g. oculoplastic surgery, corneal disease, retinal disease processes) were also identified and referred to providers where available.

The scope of work was discussed with laboratory partners, given the technical challenges potentially associated with a small volume of ocular fluid sample that was anticipated for molecular testing of EBOV RNA by RT-PCR. Additional testing that for other infectious etiologies of uveitis that was not widely available in Sierra Leone included serologic testing for Ebola IgG, rapid plasma reagin (RPR), and HIV. Molecular pathologic evaluation was facilitated by the United States partners, necessitating Sierra Leone government approvals (i.e. Chief Medical Officer, Pharmacy Board, Office of National Security), along with Centers for Disease Control and Prevention import permits and appropriate Materials Transfer Agreements between institutions to facilitate transport of laboratory specimens. Other international partners who were in-country during the EVD outbreak (i.e. China Centers for Disease Control) also provided technical guidance for the study design, as well as capacity for additional immunologic evaluation of EVD survivors in the EVICT Study. Table 1 summarizes the key stakeholders, roles of each of the multi-national partners, and key processes of the EVICT program.

**Table 1:** Overview of EVICT study implementation, host and visitor country considerations*.

Key Process of Clinical Research and Goals (Bulleted)	Stakeholders involved/Steps Taken
Definition of the patient care question and approach Identify the problem and impact (i.e. Ebola virus persistence in patients needing cataract surgery) Outline the approach to patient care	Patients–Ebola survivors and close-contacts of Ebola survivors with potential exposure Ministry of Health and Sanitation National Eye Program ophthalmologists, eye surgeons and mid-level providers Emory Ophthalmology, Infectious Disease specialists Partners in Health and multiple non-governmental organizations (NGOs) World Health Organization and public health experts Centers for Disease Control and Prevention
Ethics and Institutional Review Board approval Independent review of benefit of study, safety, and informed consent process Host in-country and external approvals Annual review to ensure amendments, consents and protocol up-to-date	Sierra Leone Ministry of Health and Sanitation Ethics and Institutional Review Board Committee Emory University Institutional Review Board
Partnership Alignment Programmatic support Identification of patient populations and need Patient mobilization Technical guidance and expertise Public health awareness and guidanace Guidance for patient sensitivity, study awareness, and sociocultural considerations	Ministry of Health and Sanitation National Eye Programme, Sierra Leone Ministry of Health and Sanitation Chief Medical Officer, District Medical Officers, and medical leadership Sierra Leone Association of Ebola Survivors/Survivor Health Advocates Emory Ophthalmologists, Infectious Disease specialists World Health Organization Global Outbreak Alert Response Network Lead NGO–Partners in Health Multiple NGOs involved in Ebola response–MSF, GOAL, Save the Children
Laboratory Alignment Ensure accurate testing to answer clinical research questions related to patient care Quality control for specimen handling (i.e. infectious pathogen transport, temperature regulation)	Kenema Government Hospital Lassa Fever laboratory Centers for Disease Control and Prevention (Materials Transfer Agreement for Import/Export and laboratory guidance) Kissy United Methodist Church in-hospital laboratory China Centers for Disease Control and Prevention United States Army Medical Research Institute of Infectious Diseases (US AMRIID)
Site Selection and Approval Verification of adequate space for procedure, ocular fluid sample handling and storage Verification of site for infection prevention and control precautions Local hospital, medical board, and institutional approvals prior to study	Ministry of Health and Sanitation National Eye Programme Ophthalmologists Lowell and Ruth Gess Eye Hospital leadership Kissy United Methodist Church Medical Board Non-government organization guidance and support (i.e. Central Global Vision Fund) Emory University Infectious Disease and Ophthalmology service verification of site and study design
Patient Mobilization and Program Implementation Ensure adequate patient recruitment Address patient clinical service needs Promote understanding of research protocol, clinical need, potential benefits and risks to Ebola survivors Communicate program and support informed consent for patients with adequate interpretation	Ministry of Health and Sanitation, Sierra Leone–Comprehensive Program for Ebola Survivors Sierra Leone Association of Ebola Survivors and Survivor Health Advocates Partners in Health and NGOs involved in Ebola outbreak response
Preoperative Ophthalmic Examination, EVICT Study Procedures Design workflows for high-volume EVICT patient screening in urban and rural settings Conduct visual acuity, ophthalmic exam and diagnostic procedures Perform ocular fluid sampling for EVD survivors Perform cataract surgery using manual small incision cataract surgery Provide opportunities for education for local mid-level providers, and ophthalmologists from Sierra Leone and Guinea Ensure long-term follow-up for patients following cataract surgery	Ministry of Health and Sanitation Sierra Leone ophthalmologists and mid-level providers Ophthalmologist based at Lowell and Ruth Gess Eye Hospital Research administrative lead from Lowell Ruth Gess Eye Hospital Ophthalmic technicians from Lowell and Ruth Gess Eye Hospital Emory University ophthalmologists and partners Emory University Infectious Disease physicians for IPC monitoring during study procedure Kissy United Methodist Church Hospital anesthesia technician Other study personnel: Hygienist, Phlebotomist Visiting ophthalmologists from Guinea

*Key process steps of clinical research and their goals are shown. These steps involved stakeholders in-country and visitor country organizations and institutions involved in implementing the clinical research project. Steps included definition of key patient needs and approaches, ethical and regulatory approvals, partnership alignment, laboratory alignment, site selection, patient mobilization, patient protection, and sociocultural context for proper program implementation of the technical demands of the project (i.e. patient evaluation, procedures, surgery, and ensuring appropriate long-term follow-up).

### Ethics and institutional review board approvals

Ethics and Institutional Review Board approvals were obtained from the MOHS and Emory University for the EVICT Study. Participants provided written informed consent after a thorough discussion of the risks, beneftis, and alternatives to participation including the key point that their ophthalmic care would be provided regardless of participation in the EVICT study. An opportunity to ask questions through an interpreter was given prior to obtaining consent to ensure consent. Consent was obtained for minors less than 6 years old via written consent from parents or guardians. Verbal assent was obtained for children 6–10 years old in addition to parent/guardian assent, and written assent was obtained for children 11–17 years old in addition to parent/guardiain assent, consistent with Emory University and MOHS Ethics and Institutional Review Board policies. The participants in this manuscript have given written informed consent (as outlined in PLOS consent form) to publish case details and the clinical environment.

A memorandum of understanding and detailed work plan between key partners was drafted to ensure clear resource management, organization and assurance of tasks to be completed. Given that EVD survivors are considered a vulnerable population, multiple conversations with SLAES leadership were helpful to properly communicate the goals of the study, which were driven by the clinical need, the uncertainty surrounding the question of whether EBOV could persist in the eye at long-term follow-up, and impact of reporting the clinical outcomes and scientific findings. The information gathered from assessing ocular fluid for Ebola viral persistence prior to cataract surgery would potentially inform guidance for ophthalmic care for future EVD outbreaks. During the meetings with SLAES leadership, these ideas were communicated, particularly related to the importance of ocular fluid sampling prior to cataract surgery, postoperative follow-up and study design.

### Patient eligibility, recruitment and mobilization

The Sierra Leone Ministry of Health National Eye Screening Program for Ebola Survivors, Partners in Health, and the Sierra Leone Association of Ebola Survivors (SLAES) were the key partners for the recruitment of patients in the EVICT Study. Patients with vision impairment and were diagnosed with cataract through the National Eye Screening program were offered an EVICT screening evaluation [20]. In addition, any EVD survivor who was known to the SLAES organization as having symptoms of cataract (i.e. blurred vision, glare, haloes, or loss of color vision) was eligible for a screening evaluation. A full dilated ophthalmic evaluation was performed by a trained ophthalmologist, and EVD survivors were offered enrollment if they met inclusion criteria. Details of the evaluations and site design have been described previously [15].

The ocular fluid sampling and cataract surgery were offered in two phases primarily due to logistical challenges surrounding transportation. In Phase 1, survivors were recruited primarily from major urban areas and regions with a high EVD burden and included Western Area Urban, Western Area Rural, and Port Loko. In Phase 2, EVD survivors were screened from outlying rural districts outside the original areas of recruitment (e.g. Bombali, Tonkolili, Pujehun, Kenema and others).

### Study site and design: Leveraging infectious disease, public health, and ophthalmology expertise for infection control

Site selection of the Lowell and Ruth Gess Eye Hospital (LRGEH) involved partnered decision-making and approvals by the Kissy United Methodist Church LRGEH Medical Board, Central Global Vision Fund, and Christian Blind Mission International, and each played a key role in the infrastructural maintenance, personnel and operations of the study sites. The impact of the study for EVD survivors and the diligent preparations needed for patient care, and adherence to infection control principles for all of the stakeholders were emphasized. The overall site and ophthalmic procedure room design for infection prevention and control was developed with Emory and West African ophthalmologists, public health experts from the WHO, and the Emory University Serious Communicable Disease Unit infectious disease physicians (S1 File). In addition, we utilized WHO Guidelines in EVD patient care for infection control precautions [21].

**Fig 1:**
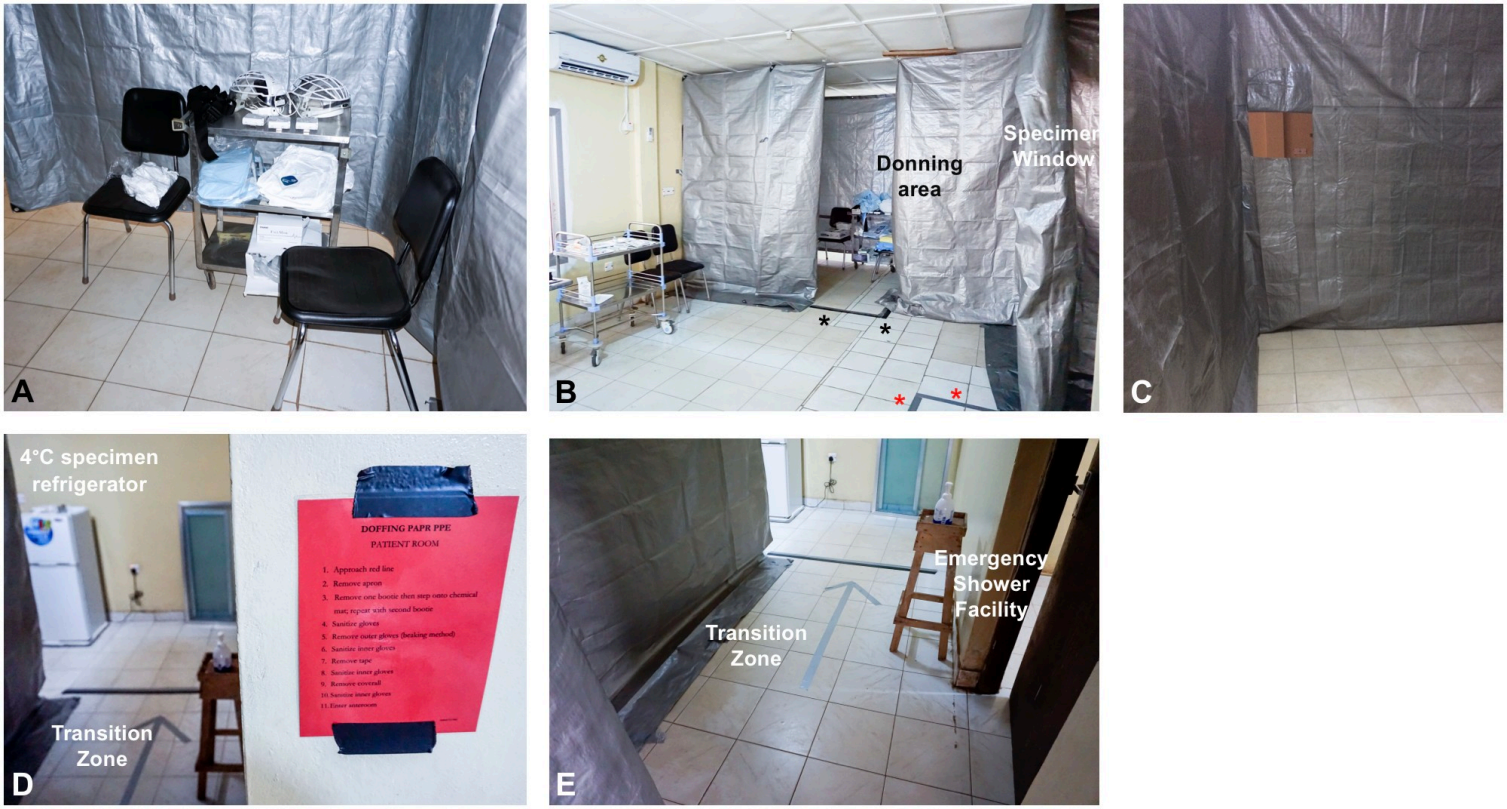
Photograph composite of EVICT study site. The EVICT study site was prepared with input from the Ministry of Health and Sanitation National Eye Programme, Sierra Leone input, along with guidance from Emory Ophthalmology, Infectious Diseases specialists, World Health Organization, and public health officials. A donning station was designed with powered air purifying respirator (PAPR) hoods, fluid impervious Tyvek suits, and shoe covers at the entryway to the procedure rom (A). The procedure room created from tarp partitions hooked from the ceiling, contained only the minimum necessary supplies for each ocular fluid sampling procedure with visibility to the donning area, marked by duct tape (B, black asterisks). A specimen window is on the wall of the tarp and duct tape (red asterisks) marks the doffing area for providers to exit via the transition zone. A specimen window is open to a clean area where ocular fluid samples are passed after double-bagging and disinfection of the specimen container (C). Visible signage for doffing is labeled and visible at the transition zone with a duct tape large arrow (D) with access to hand disinfection in the transition zone (E). An emergency shower facility is available in the event of contamination. Photographs A-D courtesy of Mr. Andrew Gess.

For the ophthalmic procedure suite, a unidirectional flow design was implemented so that donning could occur prior to entry and PPE doffing could be performed and monitored prior to exit via a transition zone (Fig 1). In each area, a physician trained in high-level PPE for the care of EVD patients monitored donning, performance of the ophthalmic procedure, and doffing. Alcohol-based hand sanitizer was present in multiple locations to ensure proper disinfection, particularly when handling ocular specimens that potentially contained infectious material.

### Personal protective equipment

The PPE for physicians, patients, and assistants was determined by the specific risk level for EBOV exposure, proximity to the procedure site, and the splash potential. For example, the ophthalmologists who were performing anterior chamber paracentesis donned full PPE for the procedures. These included a PAPR mask, fluid impervious Tyvek suit with shoe covers, and surgical gloves at all times. PPE for patients included a bouffant or surgical cap, apron and shoe covers. Observers in the room stood approximately six feet away from the table on the contralateral side of the patient to enable procedure observation while maintaining a safe distance during the procedure and after specimen transfer. The infectious disease physicians wore a bouffant cap or surgical cap, protective eyewear, aprons, shoe covers and gloves for specimen transfer through a specimen window (Fig 1).

### EVICT ophthalmic screening visit

**Fig 2:**
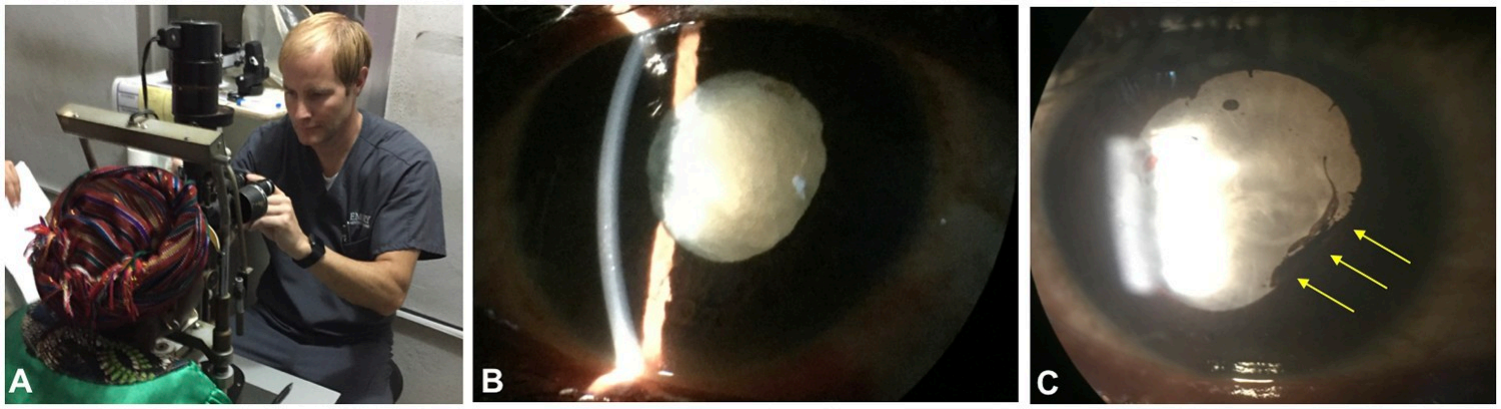
Slit lamp photography and high quality iPhone photographs through slit lamp oculars. Ophthalmic screening for cataract included evaluation of visual acuity, intraocular pressure measurement, slit lamp examination, dilated funduscopic examination and ultrasonography. While advanced photography was largely unavailable, photography could be performed with an iPhone and the ocular of the microscope (A). Resultant iPhone photographs through the ocular of the microscope showed white mature cataract (B, C) and posterior synechiae in eyes of Ebola virus disease survivors indicating chronic untreated inflammation (C, yellow arrows). High-resolution photography through operator stabilization enables visualization of anterior capsule crenations and cortical clefts within intumescent cataract.

Because of the volume of patients who were screened, a system of vision examination was developed in modular stations. The full ophthalmic examination was divided into modular stations, allowing for a design of the space, expertise, and equipment necessary to carry out screening operations with basic ophthalmic equipment (Fig 2). The stations included 1) History-taking; 2) Visual Acuity; 3) Ocular Vitals (Pupil, Motility, Visual Fields) where patients are also dilated; 4) Slit lamp examination and dilated examination with indirect ophthalmoscopy. These stations provide a way to segregate the portions of the exam both for clinic flow and opportunities for teaching (e.g. learner(s) paired with an ophthalmologist/examiner at each station). Depending on the numbers of fully trained personnel and equipment available, these stations can be modified for both mobile clinics and free-standing eye centers and clinics.

For patients who had a visually significant cataract (i.e. impeding activities of daily living or work), enrollment into the EVICT Study was offered. The complexity of the consenting process, ophthalmic examination, and ocular fluid sampling procedures required a coordinated effort on separate study days (i.e. clinic days for consenting and examination; procedure days for ocular fluid sampling, storage, and organization of patient samples for distant shipment). The ophthalmic examination and study screening were performed at the beginning of the week followed by ocular fluid sampling at a scheduled date. Once a negative EBOV RT-PCR result was received, the patient could proceed with cataract surgery, which occurred at a later date.

### Procedure day setup

The procedure day involved patient processing through several stations prior to the procedure and post procedure monitoring, including (consent and counseling), phlebotomy station, followed by the ocular fluid sampling, and post procedure observation.

### Consent and counseling

During consent and counseling, it was vital to have interpreters available for a discussion of risks, benefits, and alternatives to the protocol enrollment and performance of the procedure. In addition, patients were given the opportunity to ask questions related to any part of the procedure and voice understanding. Interpretation involved individuals who could speak one of several languages (Krio, Mende, Temne, and Limba). However, in rare situations a dual interpreter system was needed (e.g. English to Temne; Temne to one of several dialects). During the counseling and consent process, the environment where the patients would have their procedures performed was also discussed. Specifically, an EVD survivor who previously had undergone ocular fluid sampling counseled the EVD survivors that the physician would be wearing PPE for safety of everyone. This discussion helped to reduce potential post-traumatic symptoms, anxiety, or fear related to the patient’s prior hospitalization in an Ebola Treatment Unit. After the extensive counseling process, a sticker for identification was placed above the ocular procedure site and later verified in the procedure room. The consent process also included an assent process for children between 13 and 18 years old approved by Sierra Leone and Emory IRBs.

### Phlebotomy and laboratory assessments

Following the full informed consent process, the patient was escorted to the phlebotomy area with an interpreter, as well as a guardian when needed. Peripheral blood was collected for serological testing, including Ebola IgG and IgM to confirm EVD survivor status, Lassa IgG and IgM, and other etiologies of uveitis (RPR, HIV), as well as for immunologic assays. Laboratory assessments were limited due to lack of reagents for other diseases that could also be associated with uveitis (e.g. testing was not performed for sarcoidosis with ACE and lysozyme).

### Ocular fluid sampling/Anterior chamber paracentesis and aspiration

The personnel involved in the procedure included two Uveitis and Retina fellowship-trained ophthalmologists (i.e. for performance of the procedure and assistance), an infectious disease physician for monitoring donning and doffing PPE and specimen handling, and observers, that included eye care providers/health care workers from both Sierra Leone and Guinea. The details of the procedure have been described previously but included ocular surface conjunctival pre and post anterior chamber paracentesis and aqueous humor or vitreous fluid sampling [15].

**Fig 3:**
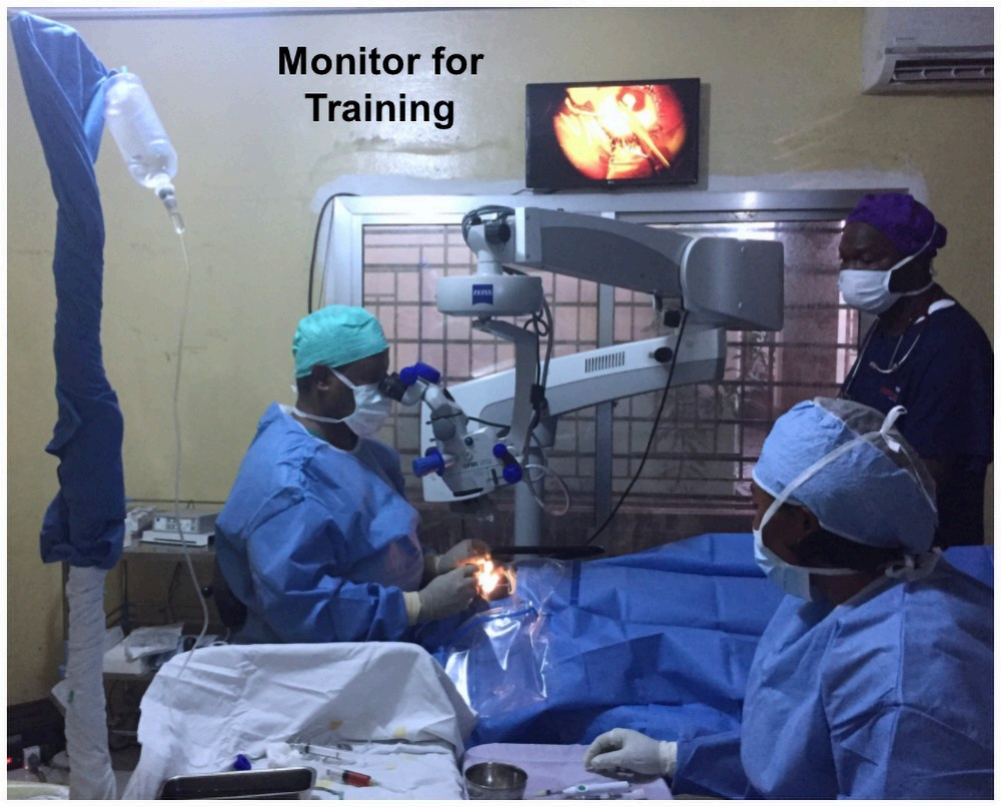
EVICT cataract surgery room setup. Aqueous humor and vitreous procedures were performed with an operator and assistant in full personal protective equipment (PPE). Infectious Diseases specialist monitoring was essential to ensure proper infection prevention and control precautions during the procedure, with specimen handling and during the donning and doffing of PPE. Sierra Leone observers and technical staff in PPE were also present for interpretation and observation of the procedure for training purposes. Following the ocular fluid sampling procedure, once Ebola virus RT-PCR testing of aqueous humor yielded negative results, manual small incision cataract surgery MSICS) could be performed in surgical PPE as shown. A video monitor was attached to the surgical microscope for additional education and teaching purposes.

One of the challenges for the procedure was the available dim lighting. To improve stereopsis for the ocular fluid sampling procedure, a penlight shined at an approximately 30 degree angle from the horizontal midline was sufficient for the operator to visualize the anterior chamber depth for needle placement to avoid inadvertent trauma to the lens and the anterior surface of the capsule enveloping the cataractous lens (Fig 3).

Specimen handling included strict infection prevention and control precautions with double-bagging and external disinfection of all biospecimen containers. Following transfer of the specimen through a specimen ‘window’ that was 2 feet by 2 feet square cut out of the tarp (Fig 1). The specimen receiver, who was also gloved, handled the specimen, disinfected the bag and placed the specimen in cold chain storage, and also recorded key observations during the procedure (i.e. volume of fluid obtained, complications). Following the procedure completion, the eye was irrigated free of anti-septic solution and an eye patch and eye shield were applied. The patient exited through the transition zone where their hands were disinfected prior to removal of their PPE. For health care provider doffing, this process was monitored by the ID physician to ensure that no exposure occurred. This included chlorine spray disinfection of the gloves and disinfection after removal of shoe covers. The health care provider walked through a mat immersed in chlorine before exiting. All other individuals involved in the procedure followed appropriate IPC precautions during the exit from the procedure room.

### Specimen handling and transfer

All specimens in their container and biohazard specimen bags were placed in a 4° temperature-monitored refrigerator. Because the receiving laboratory (i.e. Kenema Government Hospital Lassa Hemorrhagic Fever laboratory) was four hours away from the LRGEH site, the specimens were refrigerated overnight at a 4° temperature-monitored refrigerator prior to pick-up by a medical courier trained in specimen delivery of highly infectious pathogens. All specimens were stored in a dual container system with ice packs (i.e. double-bagged and stored in a medical cool box to ensure cold chain transport).

### Ebola virus RT-PCR testing

Following receipt of the conjunctival swab specimens, aqueous or vitreous humor specimens, a member of the ophthalmology team was contacted by the KGH laboratory team to confirm receipt of the samples. The assays performed consisted of an in-house assay for EBOV RT-PCR with primer targets that have been described previously [22]. Laboratory results were communicated to the ophthalmology care team; following confirmation of a negative EBOV RT-PCR result, the patient underwent manual small incision cataract surgery (MSICS).

### Manual Small Incision Cataract Surgery (MSICS)

MSICS procedures were performed by an in-country ophthalmologist with PPE and infection prevention and control guidance from infectious disease specialists. The cataract surgery was performed in a room designated for infection control purposes (i.e. not in the main operating theater) educational observation, and specimen collection (Fig 3). Although ocular specimens (conjunctiva, aqueous humor) collected in phase 1 of the EVICT study tested negative for EBOV by RT-PCR, whether the cataract lens, lens capsule or vitreous could have EBOV persistence was unknown. For this reason, handling of these specimens as well as aqueous humor sampling was performed with IPC precautions as in Phase 1 of the study. Specifically, after any specimen was harvested from the eye (i.e. ocular fluid, lens capsule, cataract lens), the syringe (i.e. for ocular fluid) or container with the specimen formalin (i.e. for tissue specimens) was wiped down with hospital grade disinfectant and then double-bagged for transport to the KGH laboratory.

### Postoperative follow-ups

Patients were evaluated at postoperative day 1 and at scheduled intervals (i.e. 1 week, 2 weeks, 1 month, 3 months and 5–6 months). For patients with substantial ocular inflammation identified postoperatively, more frequent follow-up was recommended. Feedback from the in-country ophthalmologist and United States-based team via teleconference or email on a bimonthly basis was implemented to communicate clinical care questions during the postoperative period. A SLAES medical representative communicated with patients regularly so that patients with new symptoms could also be evaluated by the Sierra Leone ophthalmology care team if needed.

#### Challenges and lessons learned

While there were challenges to bring together multiple partners to design, implement, and carry out this multifaceted study in a resource-limited setting, a number of lessons learned are applicable to ophthalmic research and care related to acute EVD and sequelae, as well as to other emerging infectious diseases. While implementation of clinical research protocol in *well-resourced* countries can require more time than expected due to a multiplicity of factors (e.g. delays in funding, study design development, patient recruitment, or regulatory approvals), our partnership faced additional challenges given the *resource-limited* environment (e.g. patient transportation, stable electricity and power, facility design with spatial constraints).

### Timeline

The inception of the EVICT Study planning was in October 2015, with study launch in mid-2016 and completion by mid-2018. During this time, widespread concerns about EBOV persistence in ocular fluid resulted in multiple eye hospitals ceasing all ocular surgery. Only 4 ophthalmologists were in-country to serve approximately 7 million people in Sierra Leone. Besides increasing eye care capacity through training, additional focus of ongoing work is related to research on ocular complications in EVD survivors, and eye care training, including ocular fluid sampling, and infection prevention and control. Other aspects that affected our timeline was the scarcity of laboratories performing EBOV RT-PCR and the long distance from the Lowell and Ruth Gess Eye Hospital to the KGH Lassa Fever laboratory, making same-day RT-PCR analysis not possible. Future studies could include same-day EBOV RT-PCR testing to avoid delays in surgery while awaiting a negative EBOV RT-PCR result.

### Special considerations: Pediatric patients

Cataracts, particularly in very young children, have a high rate of posterior capsular opacification, long-term risk of glaucoma, and additional challenges if a EVD survivor has had uveitis [23,24]. Because of severe inflammation that may ensue following cataract removal, patients are sometimes left without a lens (i.e. aphakic) and a capsulotomy is required. For this reason, pediatric cataract surgery is often performed by either a pediatric ophthalmologist or vitreoretinal surgeon (i.e. pars plana vitrectomy, lensectomy, and capsulotomy). Currently, no vitreoretinal surgical capacity is available in Sierra Leone, and efforts are underway to address this medical care gap. The availability of anesthesiologist support for pediatric cataract surgery also presents unique challenges to ensure adequate perioperative measures for safety.

### PREVAIL VII: Collaborating with other research programs

While the EVICT Study was ongoing in Sierra Leone, the NIH-funded PREVAIL III Study was increasingly recognizing cataract, uveitis and visual disability in their longitudinal cohort of EVD survivors in Liberia [3]. Following conversations with the NIH, John Snow, Inc, Samaritan’s Purse, and ELWA Hospital in Liberia, we initiated a multiple partnership collaboration, which employed similar principles of the EVICT Study related to facility design with unidirectional flow, infection prevention and control, a two-phased approach of ocular fluid sampling followed by cataract surgery. The encouraging twelve-month results following cataract surgery in EVD survivors in Liberia patients were recently reported [25,26].

## Conclusions

The EVICT Study provided a unique opportunity to address a timely and unanswered question related to whether EBOV may persist in the eye in EVD survivors who were awaiting vision-restorative surgery. The absence of EBOV RNA by RT-PCR provided reassurance to the eye surgeons and care team that cataract surgery could be performed in EVD survivors that there was no evidence of increased risk of Ebola virus exposure to the provider during the time points assessed. Besides these key conclusions relevant to individual Ebola survivors, the methodologies detailed in this report emphasize a circumspect approach and high level of detail required for program implementation in complex environments. Moreover, the numerous partnerships, community engagement, patient care and laboratory workflows, while tailored to the resource-limited settings, provided opportunities for collaboration in research and education for vision health systems strengthening. Lessons learned from the ophthalmic care of EVD survivors may also have broader application for invasive procedures related to immune privileged organs for other emerging infectious diseases, including those with ocular sequelae.

## Supporting information

S1 File EVICT study investigators roster. (DOCX)
