# Supplementary material for: Implementation of the Ebola Virus Persistence in Ocular Tissues and Fluids (EVICT) study: Lessons learned for vision health systems strengthening in Sierra Leone
Source: PLoS One. 2021 Jul 9;16(7):e0252905. doi: 10.1371/journal.pone.0252905 (PMC8270115; doi:10.1371/journal.pone.0252905)
Supplement: S1 File — (DOCX) [file pone.0252905.s001.docx]

**Supplementary File**

**Ebola Virus Persistence in Ocular Tissues and Fluids Study (EVICT) Investigators**

**Study Group Roster**

**Emory University,** *Atlanta, GA, USA*

Steven Yeh, MD, Jessica Shantha, MD, Brent Hayek, MD, Duncan E. Berry, MD, J. Clay Bavinger, MD, Alcides Filho Fernandes, MD, Colleen S. Kraft, MD

**Integrated Research Facility, Division of Clinical Research, National Institute of Allergy and Infectious Diseases, National Institutes of Health***, Bethesda, MD, USA*

Ian Crozier, MD

**Ministry of Health and Sanitation National Eye Program, Sierra Leone,** *Freetown, Sierra Leone*

Matthew J. Vandy, MD, Jalika Mustapha, MD, John G. Mattia, MD, Moges Teshome, MD, Lloyd C. Harrison-Williams, MD

**Ministry of Health and Sanitation, Sierra Leone,** *Freetown, Sierra Leone*

Kwame O’Neill, MD, Sarian Kamara, MD, Alie Wurie, MD

**Kenema Government Hospital Lassa Hemorrhagic Fever Laboratory***, Kenema, Sierra Leone*

Augustine Goba, John D. Sandi, Mambu Momoh, Simbirie Jalloh, Donald S. Grant, MD, MPH

**Partners in Health,** *Boston, MA, USA*

Paul E. Farmer, MD, Kerry Dierberg, MD*, Joyce Chang, RN*

**United Kingdom Public Health Rapid Support Team Public Health England/ London School of Tropical Medicine,** *London, United Kingdom*

Daniel G. Bausch, MD, MPH

**Tulane University School of Medicine,** *New Orleans, Louisiana, USA*

Robert F. Garry, PhD, Jessica N. Hartnett, PhD, Jeffrey G. Shaffer, PhD, John S. Schieffelin, MD

**University of California San Francisco,** *San Francisco, CA*

Nisha R. Acharya, MD

**United States Centers for Disease Control and Prevention**, *Atlanta, GA, USA*

Timothy M. Uyeki, MD

**Central Global Vision Fund,** *Milbank, SD, USA*

Roger Reiners, Melanie Reiners, Lowell A. Gess, MD

**Sierra Leone Association of Ebola Survivors*,*** *Freetown, Sierra Leone*

Mohamed Mansaray, Yusuf Kabba, Daddy Kamara

**University of Toronto,** *Toronto, ON, Canada*

Sharmistha Mishra, MD, Adrienne K. Chan, MD, Rob Fowler, MD

**University of Liverpool,** *Liverpool, United Kingdom*

Tim O’Dempsey

**National Institute for Viral Disease Control and Prevention, Chinese Center for Disease Control***, Beijing, China*

William J. Liu, PhD

**World Health Organization,** *Geneva, Switzerland*

Faiqa K. Ebrahim, MD*

**Comprehensive Programme for Ebola Survivors***, Freetown, Sierra Leone*

Taylor Hendricks*, Erick Kaluma*

**United States Army Medical Research Institute of Infectious Diseases (US AMRIID)***, Frederick MD, USA*

Sina Bavari, PhD, Gustavo Palacios, PhD

*Transferred institution since study inception
